# Comparison of two alternative study designs in assessment of medicines utilisation in neonates

**DOI:** 10.1186/1471-2288-14-89

**Published:** 2014-07-16

**Authors:** Georgi Nellis, Irja Lutsar, Heili Varendi, Karolin Toompere, Mark A Turner, Jennifer Duncan, Tuuli Metsvaht

**Affiliations:** 1 Institute of Microbiology, Tartu University, Tartu, Estonia; 2 Neonatal Unit, Children´s Clinic, Tartu University Hospital, Tartu, Estonia; 3 Department of Public Health, Tartu University, Tartu, Estonia; 4 Department of Women’s and Children’s Health, Institute of Translational Medicine, University of Liverpool, Liverpool, UK; 5 Neonatal Unit, Liverpool Women’s NHS Foundation Trust, Liverpool, UK; 6 Research and Development, Alder Hey Children's NHS Foundation Trust, Liverpool, UK; 7 Paediatric Intensive Care Unit, Clinic of Anaesthesiology and Intensive Care, Tartu University Hospital, Tartu, Estonia

**Keywords:** Pharmacoepidemiologic methods, Cross-sectional studies, Data collection, Drug/excipient exposure

## Abstract

**Background:**

Estimates of prevalence are known to be affected by the design of cross-sectional studies. A pan-European study provided an opportunity to compare the effect of two cross-sectional study designs on estimates of medicines use.

**Methods:**

A Service evaluation survey (SES) and a web-based point-prevalence study (PPS) were conducted as part of a European study of neonatal exposure to excipients. Neonatal units from all European Union countries plus Iceland, Norway, Switzerland and Serbia were invited to participate. All medicines prescribed to neonates were recorded during three-day and one-day study periods in the SES and PPS, respectively. In the PPS individual demographic and prescription data were also collected.

To compare the probabilities that a particular medicine would be reported by each study multilevel mixed effects logistic regression models with crossed random effects were applied. The relationship between medicines exposure at the unit and individual levels in the PPS data was assessed using polynomial regression with square root transformation.

**Results:**

Of 31 invited countries 20 and 21 with 115 and 89 units joined the SES and PPS, respectively. Out of 5,572,859 live births in invited countries in 2010 a higher proportion was covered by units participating in the SES compared to the PPS (11% vs 6%, respectively; OR 1.89; 95% CI 1.87-1.89). A greater number of active pharmaceutical ingredients (API), manufacturers and trade names were registered in the SES compared to the PPS. High correlation between the two studies in frequency of use for each specified API was seen (R^2^ = 0.86). The average probability of a department to use a given API was greater in the SES compared to the PPS (OR 2.36; 95% CI 2.05-2.73) with higher frequency of use and longer average duration of prescription further increasing the difference. The polynomial regression model described the correlation between APIs exposure on unit and individual level well (R^2^ = 0.93).

**Conclusion:**

The simple data structure and longer study period of the SES resulted in improved recruitment and higher likelihood of capture for a given API. The frequency of use at the unit level appears a good surrogate of individual exposure rates.

## Background

Newborn babies receive a range of medicines many of which have not been studied adequately. Furthermore, exposure to medicines introduces risks and risk assessment requires information about the extent of medicines use. One neglected source of risk is the inclusion of excipients in medicines. Thus there is a need to gather information about medicines use in a way that captures data about active pharmaceutical ingredients (API) and the excipients. Variability in excipient content between formulations of the same API needs to be considered.

Cross-sectional studies are attractive for studies of medicines use [1-7]. In principle, data can be collected at unit or individual level. Unit level data involve listing all the products used on a unit over a specified period of time. These data are simple to collect and can be integrated with processes for medicines supply (such as sales or dispensing activity). Unit level data indicate which medicines are used (including how many distinct products, APIs and excipients are used) and will indicate whether there are geographical differences in medicines use. In combination with demographic data about the units this method can provide indicative estimates of market size. Individual level data require an extra step of data collection. This approach captures denominator data and generates more precise estimates of market size while allowing for stratification according to important clinical variables such as age of the baby. Point prevalence studies (PPS) have been used in antibiotic consumption studies [4,5,8]. Their extremely short duration may underestimate exposure to less frequently used medicines. Unit level studies can have a longer duration but may not be manageable in a multinational setting [9]. Although a recent analysis has described the impact of cross-sectional study designs on disease incidence/prevalence estimates [10], little is known about the effects of study design in research about medicines use. We are not aware of any head to head comparison of different methods and thus the question how to navigate between different methods in medicine exposure research remains unanswered.

Within the European Study of Neonatal Excipient Exposure (ESNEE) we planned to use both unit and individual level data collection in order to maximise yield for two different study questions. [11]. This gave us the opportunity to compare the two study designs – a short duration PPS and longer lasting service evaluation survey (SES). We used APIs as the unit of analysis in this methodological study.

Accordingly, we aimed (1) to describe the implementation of each method; (2) to assess the extent of discordance between the methods by comparing the probabilities that particular medicines would be reported by each method; (3) to explore the correlations between medicines exposure at unit and individual levels.

## Methods

The SES and PPS were performed as multicentre observational surveys of routine clinical practice. During the SES all medicines prescribed to neonates (≤28 days of postnatal age) were recorded on pre-formulated Excel spreadsheets over three consecutive days within a fixed period of time from May 30th to September 30th, 2011. PPS data collection was performed in a web-based database within one day during one of three fixed two-week study periods from January to February; March or May to June, 2012. Each participating unit was free to choose the most appropriate day(s) for data collection. In both surveys printable data collection forms were provided as an alternative to electronic data insertion.

### Participating units and study population

The study aimed to cover all 27 European Union countries plus Iceland, Norway, Switzerland and Serbia. A network of national contact persons (Lead Contacts) was built by the ESNEE consortium. Search for national Lead Contacts was carried out through national professional/scientific societies of neonatology, paediatrics and/or perinatology as well as through personal contacts of the ESNEE consortium members or other FP-7-funded consortia (NeoMero, TINN) [12,13]. The Lead Contact was then asked to recruit as many hospitals and units providing neonatal care in the country as possible.

At the outset, a PPS sample size estimation was planned. It was intended to base this on cluster sampling analysis stratified by country, assuming conservatively a 15% neonatal admission rate of all live births [14-17] and a response rate of 50-70% of invited units as described in previous neonatal surveys [18-21]. Based on the Eurostat NUTS (or equivalent if NUTS classification not available for the country) regional distribution of the population and reported nation-wide birth rates, the number of potentially available neonatal admissions would be calculated and the representative sample size estimated for each country and region [22]. A complete list of institutions involved in neonatal care would be created for each randomly chosen NUTS2 region with subsequent involvement of all units to cover a whole region with proportional representation of different unit levels. Alternatively, if a full list of neonatal units was not available and/or the response rate in the SES would suggest critically low recruitment, all contacts provided by Lead Contacts were to be invited to participate in the PPS.

All general neonatal, intermediate and neonatal intensive care (NICU) as well as mixed paediatric and neonatal intensive care units with more than 50% of admissions consisting of neonates were eligible with stratification according to the level of care: level 1 with special neonatal care; level 2 with high dependency care, short term intensive care and low birth weight care, and level 3 with comprehensive intensive care for extremely low birth weight infants available [23,24]. Departments, offering different levels of care were classified according to the highest level they provided.

### Data collection

In both studies all eligible neonates in the unit at 8 a.m. of the study day and in the SES additionally those admitted within the next 72 h, were included. The SES recorded the number of neonates receiving any prescription during the study period. In the PPS individual demographic data including gender, gestational age (GA), birth weight, 1st and 5th minute Apgar score, current body weight, postnatal age and organ dysfunctions, were recorded for all neonates receiving any prescriptions active on the study morning at 8 a.m.

### Prescription data

All prescriptions, including micronutrients, iron, vitamins, parenteral nutrition solutions and topical agents were recorded. Blood products, glucose and electrolyte solutions, vaccines, nursery care topical agents, herbal medicines and food including breast milk fortifiers were excluded. The following information was collected for every medicine prescribed: trade name, manufacturer, API, strength, pharmaceutical dosage form and route of administration. Additionally individual dosing regimen together with prescription starting date was collected in the PPS.

Extemporaneous or compounded forms of medicines were not included in described analysis. The use of caffeine and morphine was reported differently in the two studies. UK units using ´special´ manufactured [25] preparations, rather than extemporaneous formulations used in the rest of European countries, were over-represented in the PPS. Accordingly, these two APIs were excluded from the analysis.

In both studies the data were pooled for analysis without any comment on treatment strategies of individual participating units.

### Data management and statistical analysis

SES data collection forms were checked for legibility with two rounds of queries regarding missing data (response rate 52%). Each medication was classified according to brand name, manufacturer and pharmaceutical dosage form. The list of medicines obtained in the SES was used to prepopulate choices in the PPS database. To minimize the risk of data entry errors all ´newly appearing´ medicines in the PPS were added to the database by the ESNEE team.

Statistical analysis was performed with Stata Software (ver. 12.1). Descriptive statistics were used as appropriate to describe the variability of medicine exposure in both studies.

To identify the effect size of the study method, the average probabilities of the units to use any given API in SES vs PPS were compared by multilevel mixed effects logistic regression models with crossed random effects [26]. Since the sample of departments in the two studies was partly overlapping, department and API were added to the model as a random effect when comparing the average odds of using an API between the two study methods. The outcome variable was a binary indicator showing whether an API was used in a specific unit in a specific study. The analysis was limited to agents used in more than one unit in the SES. All models were adjusted for geographic region (Northern, Southern, Western and Eastern Europe according to the United Nations Statistics department [27]) and unit level. These variables were identified as potential confounders in an exploratory population averaged Poisson regression model with robust standard errors [28] analysis including 10 most often used APIs. Furthermore the effect of unit size (described by number of annual admissions), frequency of a given medicine use (described by proportion of units using it) and the duration of the prescription (described by the average time from the start of prescription to the PPS study day) on the estimated method effect size was explored.

To study the relationship between the number of units using each API and the number of prescriptions for that API polynomial regression model with square root transformation was used based on the PPS data.

### Ethical considerations

The SES did not collect any personal data and so did not require ethics committee approval in any of the participating countries. For participation in the PPS Ethics Committee approval was obtained in compliance with the respective national guidelines. In compliance with local authorities no consent for individual patients was sought, as all data were collected in routine clinical practice and anonymised before leaving study sites.

## Results

### Characterisation of participating units

The cluster randomised approach to sample size calculation described in Methods section, was not feasible because no country had a full list of neonatal units. Accordingly, all country Lead Contacts were invited to participate in the PPS with as many units as possible. Of 31 invited countries 20 and 21 (Austria, Belgium, Bulgaria, England, Estonia, Greece, Hungary, Ireland, Italy, Latvia, Lithuania, Norway, Portugal, Romania, Serbia, Slovenia, Spain and Switzerland in both; Denmark and Poland only in the SES; Malta, France and the Netherlands only in the PPS) with 115 and 89 neonatal units joined the SES and PPS, respectively. The number of units per country varied from one to 20. In the SES compared to the PPS a higher proportion of annual live births was covered overall (11% vs 6% of 5,572,859 live births in participating countries in 2010, respectively; OR 1.89; 95% CI 1.87-1.89) and in each European region (Table 1). Regional distribution varied with higher representation of Eastern compared to Western Europe (10% and 6% of 968,910 and 1,987,904 live births, respectively; OR 1.80; 95% CI 1.78-1.81) and lower compared to Northern and Southern European countries (12% and 18% of 1,357,998 and 1,258,047; OR 0.52; 95% CI 0.51-0.52 and 0.84; 0.83-0.84, respectively) in the SES. The same regional distribution was seen in the PPS (data not shown). No difference in the prevalence of different levels of care between the two studies was observed. However, the prevalence of teaching hospitals was lower in the SES as compared to the PPS (OR 0.47; 95% CI 0.24-0.92) (Table 1). Similarly, the proportion of all annual admissions in units covered by the study was higher in the SES than the PPS (3% and 2% of 90235 and 61392 admissions, respectively; OR 1.58; 95% CI 1.48-1.68).

**Table 1:** Characteristics of participating units in the SES and the PPS

	**SES**	**PPS**	**OR (95% CI)**
No of participating countries	20	21	NA
No of participating hospitals	102	73	NA
No of participating units	115	89	NA
Median (IQR) No of participating units per country	3.5 (1.75;7.75)	3 (2;5)	NA
No (%) of teaching hospitals	62 (61%)	56 (77%)	0.47 (0.24-0.92)
Level distribution (%)			
Level 3	60	73	0.56 (0.31-1.03)
Level 2	33	21	1.84 (0.97-3.50)
Level 1	6	6	1.13 (0.35-3.69)
Total annual live births in catchment area of participating units* (year of reference: 2010)	593,001	349,465	1.89 (1.87-1.89)
Regional proportions of live births covered (%)**			
Eastern Europe	10	4	2.79 (2.75-2.82)
Northern Europe	18	8	2.55 (2.53-2.57)
Southern Europe	12	9	1.30 (1.27-1.31)
Western Europe	6	4	1.65 (1.63-1.66)
Annual number of admissions in participating units α	90,599	72,132	NA
Annual admissions coverage; mean% (SD) ¥	3.95 (2.74)	2.59 (1.48)	1.58 (1.48-1.68)
No of neonates in unit in the study period	3080‡	1382	NA
No of neonates receiving any drug prescription during the study period	2050	825	1.34 (1.18-1.53)

*Data available for 107 units in the SES and for 48 hospitals in the PPS; **calculation of proportions was based on the total number of live births in given region in 2010 (source of data: Eurostat; last update: 03.04.2013); α data available for 104 units in the SES and for 82 units in the PPS; ¥ based on data from 102 units in the SES and 82 units in the PPS where both annual number of admissions and number of patients during survey were available; ‡ data available for 107 units.

### Consumption of medicines in the PPS and the SES

More neonates in the SES received at least one drug prescription compared to the PPS (OR 1.34; 95% CI 1.18-1.53) (Table 1). In total, a greater numbers of APIs, manufacturers and trade names were registered during the SES than the PPS (Table 2). Slightly lower prevalence of enteral and higher prevalence of topical formulations was noted in the SES compared to the PPS with no difference in parenteral medicines use. In the PPS the number of prescriptions per neonate was inversely related to GA the average (SD) count per neonate being 4.53 (2.33); 3.95 (2.59); 2.68 (1.91) and 2.31 (1.93) in the GA bands of <28; 28–31; 32–36 and >36 weeks, respectively.

**Table 2:** Medicines consumption in the SES and the PPS

	**SES**	**PPS**	**OR (95% CI)**
No of prescriptions during the study period	NA	2608	NA
Median (IQR) No of prescriptions per neonate	NA	2 (1;4)	NA
No of active ingredients prescribed*	313	280	NA
No of trade names (by name, manufacturer, pharmaceutical dosage form and strength)	1065	624	NA
No of manufacturers	332	235	NA
Route of administration (%)			
Parenteral	58	59	0.96 (0.79-1.17)
Enteral	30	36	0.79 (0.64-0.97)
Topical	12	5	2.32 (1.57-3.42)

*All components in multicomponent drugs are counted separately.

### Correlation of medicine use between SES and PPS

**Figure 1:**
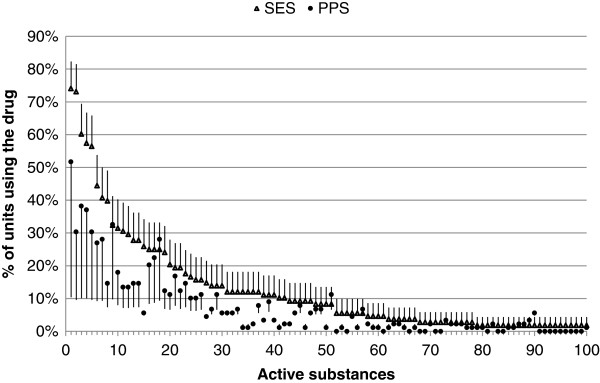
**Proportions of units prescribing frequently used active substances in the SES and the PPS.** The SES and PPS data presented as triangles and circles, respectively. Active substances that were used in more than one unit in the SES (n = 99) are presented. Each number on the x-axis identifies the individual active substance. Upper 95% CI in the SES and lower 95% CI in the PPS are shown in error bars.

Altogether 99 APIs were used in more than one unit in the SES and were included into further analysis. As shown in Figure 1 almost all APIs were more frequently used in the SES compared to the PPS but the 95% CIs were overlapping for the majority.

In multilevel mixed effects logistic regression models with crossed random effects the average probability of the departments to use each of the most common API was higher in the SES compared to the PPS (OR 2.36; 95% CI 2.05-2.73; p < 0.0005). The frequency of use and average duration of prescription further increased the likelihood of being registered in favor of the SES by 1.01 times (95% CI 1.01-1.02; p < 0.0005) and 1.02 times (95% CI 1.00-1.05; p = 0.047) per each additional percent in frequency and day in duration of prescription, respectively. Size of the department did not influence the capture probability (OR 0.99; 95% CI 0.99-1.00; p = 0.652).

**Figure 2:**
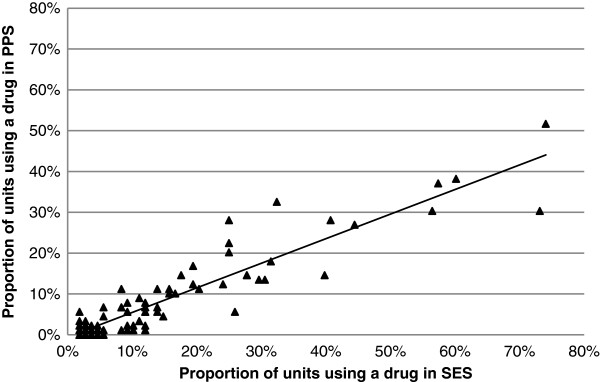
**Correlation in the frequency of medicine use between the SES and the PPS on unit level.** Correlation between the SES and the PPS for active substances used in more than 1 unit in the SES is shown. Trendline for 99 active ingredients: y = 0.6019x – 0.0053; R^2^ = 0.8605.

As shown in Figure 2 a high correlation in the frequency of medicine use between the SES and the PPS was observed (R^2^ = 0.86).

**Figure 3:**
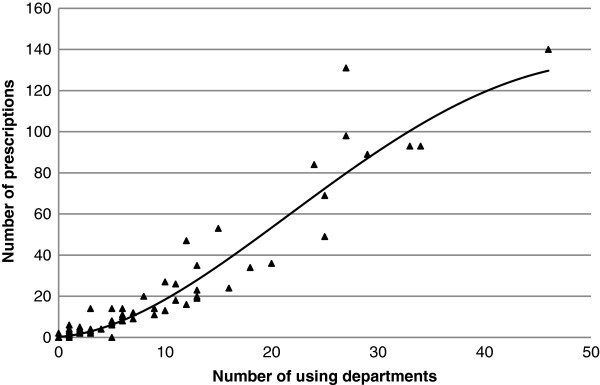
**Correlation between medicines exposure at unit and individual levels.** Number of units using each specified active ingredient (n = 99) in relation to the number of prescriptions in the PPS was observed. Polynomial regression trendline for active ingredients used in more than one unit in the SES is shown. √number of prescriptions = 0.421 *(number of departments) – 0.004 *(number of departments)^2^ + 0.485; R^2^ = 0.93.

We next evaluated the correlation between the number of prescriptions per given API and the number of units using that API in the PPS. As shown on Figure 3 there was a high correlation between these two variables in polynomial regression model with square root transformation (R^2^ = 0.93).

## Discussion

By applying two different study designs in comparable target populations we have demonstrated that simple data structure and longer study period used in the SES improved recruitment and the likelihood of capture of medicines consumption. The probability of capture for a given API was further enhanced by higher frequency of use and longer duration of prescription. This meant that the SES gave a more comprehensive list of trade names, APIs and manufacturers than the PPS. The list of most frequently used APIs was overlapping in 90% with high correlation of use frequencies between the two studies.

The strength of both methods described here is the simplicity and uniformity of data collection. A two-step approach, as used in the ESNEE project may allow simultaneous coverage of both unit-level and individual-level data in neonatal excipient exposure studies, where hardly any multi-country data are available. We have recruited so far one of the largest international cohorts of neonatal units from 21 European countries revealing a surprisingly wide list of medicines. The number of trade names applied in NICU setting was also high. The response rate of 71% on country level as well as the number of prescriptions per neonate [9,29] are similar to those reported previously [18-21]. Against the background of rising safety concerns of neonatal medicine/excipient exposure, the wide trade name list from the SES allows identification of substitution possibilities, while individual exposure data from the PPS provide a more precise quantification of the problem.

Different study designs can be applied in pharmacoepidemiology [30,31]. Cohort studies have the advantage of allowing data collection over prolonged time periods which is more likely to capture rare exposures. However, the expense and duration make it hard to implement cohort designs in multinational studies [9]. Case–control studies allow rich data collection when a limited number of medicines/excipients are targeted and data collection is limited to a few centers. The PPS and SES, in contrast, are easy to perform in multinational settings. They are time and resource saving and to some extent cover each other`s shortcomings like under- or overestimated exposure rates of rarely used medicines or need for individual data [30]. Alternately, nested study designs with longitudinal data collection allow linkage between different datasets and may prove the best option. However, to the best of our knowledge, it has hardly been used in paediatric pharmacoepidemiological studies.

When planning medicine/excipient exposure studies the research question to be answered needs to be balanced against the implications of study design. We recognize that a 3-day survey is actually very short in duration. However, even this small longitudinal component resulted in a more comprehensive list of medicines due to the better recruitment when simple data structure was used. On the other hand, when individual exposures of frequently used medicines are targeted, the increasing data volume (both structure and duration) needs to be weighed against evolving decrease in compliance that may lead to underestimation of prevalence [32]. It has been shown that the longer the recall period, the greater the imprecision (especially underestimation) of prevalence estimates [33,34], although this finding may be more relevant in non-medical informants [10]. Furthermore, not only optimal recruitment but also sample size maintenance over the course of a study becomes an issue [35].

Additionally, economic evaluation plays a role in the choice of a specific study design. There is considerable overlap between acceptable methodologies and those sanctioned by health economists [36]. Although pharmacoeconomic analysis remains beyond the scope of this study, a few comments can be made. There were no major cost-differences in conducting these studies with the exception of those associated with the development of the PPS database. Web-based collection with automated control of data completeness was applied to improve compliance and quality of reporting. We believe that wherever sufficient to answer the research question, simple data structure is favourable in terms of resource allocation. However, when a more complex format is required, reasonable additional expenditure on data collection tools may result in an improved cost-benefit ratio.

We have also shown a high correlation between APIs exposure on unit and individual level. This should allow an indirect individual exposure assessment from less demanding approach like SES under the condition of limited resources. We realize, that the estimates in the frequency of medicine use in the PPS are systematically lower compared to the SES. However, knowing the relationship between the two an estimate for one can be extrapolated from the other. Nevertheless, such calculations should be applied with caution and to estimate the exposure only for the studied population as a whole. In neonatal studies stratification by GA age allows a more meaningful risk assessment due to the strong dependence of the maturity of metabolic pathways on the postmenstrual age of the newborn [37,38]. It has been shown previously and also by us that medicines utilization pattern in extreme prematurity is different from that near or at term [9,29,39,40]. Therefore, the PPS approach should be preferred to obtain individual exposure estimates in neonates of different GA.

Some limitations of the present study need to be noted. First, the quality of data collection was dependent on the personnel entering the data. In both studies data insertion was done by medical specialists according to standardized protocols. As for the quality of reported data, in the SES most queries concerned demographic data; only a few queries were related to the information about medicines. Second, neonatal units were given the option of selecting the most appropriate day(s) for data collection. This could lead to underestimation of medicine use because less busy day(s) probably were more likely to be chosen. However, we believe that eventually this approach captured information on a larger number of neonates and medicines through improved compliance and a larger number of participating units. Third, the slightly different representation by country required exclusion of two APIs from final analyses due to differences in regional handling/approach. Fourth, the SES and the PPS were done at different times. Nested design with detailed individual data collection (PPS) performed as part of the SES was not feasible, as an interim between the two studies was needed to prepopulate the PPS database from the SES list of medicines. This could potentially reduce the overlap between the studies. In that situation our data about overlap represent a “worst case scenario”. The true concordance in API reporting may be higher than described by us. Finally, about two thirds of the 31 invited countries agreed to participate with relative inhomogeneity of the regional distribution of participating units. Although the inability to randomly select hospitals probably caused overrepresentation of teaching hospitals (61% and 77% in the SES and PPS, respectively, compared to less than 10%, reported in national hospital statistics [41]), data on the distribution of the levels of neonatal care will allow appropriate adjustments. Both SES and PPS have achieved representative sample size on patient level, the margin of error being 1.73% and 2.63%, respectively (CI 95%; response distribution 50%; population size 5.4 million live births). However, it would be valid under the condition of randomisation that remained unfeasible in our studies. Still, the current analysis gives a notion of good coverage of the target population in the SES and PPS.

## Conclusions

The less demanding approach of the SES allows better recruitment of units for longer periods resulting in a more comprehensive list of medicines and trade names. As we have shown, the frequency of use on unit level appears a good surrogate of individual exposure rates. However, more detailed information can be collected in a PPS. Investigators need to balance the advantages of a PPS with the risk of bias, which depends on the frequency as well as duration of medicine use. Simultaneous use of both methods with merged data analysis will likely result in optimal coverage of both aspects of the problem.

## Abbreviations

API: Active Pharmaceutical Ingredient; ESNEE: European Study of Neonatal Excipient Exposure; GA: Gestational Age; NICU: Neonatal Intensive Care Unit; NUTS: Nomenclature of Territorial Units for Statistics; PPS: Point Prevalence Study; SES: Service Evaluation Survey.

## Competing interests

The authors declare that they have no competing interests.

## Authors’ contributions

GN participated in the design and conduct of the study and writing the manuscript. IL participated in the design and coordination of the study and drafting/revising the manuscript. HV participated in the design and conduct of the study. KT performed the statistical analysis. MT conceived of the ESNEE project, and participated in its design and coordination, and has been involved in revising the manuscript. JD participated in the design and conduct of the study. TM participated in the design and conduct of the study and has been involved in drafting and revising the manuscript. All authors read and approved the final manuscript.

## Pre-publication history

The pre-publication history for this paper can be accessed here:

http://www.biomedcentral.com/1471-2288/14/89/prepub
